# The effects of conscious movement investment on inhibiting a simple response

**DOI:** 10.3389/fpsyg.2024.1365420

**Published:** 2024-08-13

**Authors:** Yihong You, Wan-Chun Weng, Gita Benefita Suprianto, John van der Kamp

**Affiliations:** Department of Human Movement Sciences, Faculty of Behavioural and Movement Sciences, Vrije Universiteit Amsterdam, Amsterdam, North Holland, Netherlands

**Keywords:** inhibition, ego-depletion, go/no-go task, stop-signal task, conscious movement investment

## Abstract

The relationship between a performer’s conscious involvement or investment in movement control and monitoring and the ability to inhibit the movement is still unclear. We conducted three experiments to investigate whether a higher inclination for conscious movement investment benefits the inhibition of a simple keypress response. In all experiments, the inclination for conscious movement investment was measured with the Movement-Specific Reinvestment Scale. In Experiment 1, participants performed the go/no-go task and conscious investment was manipulated by directing conscious attention either to the finger movement (i.e., internal focus) or to the resulting motion of the key (i.e., external focus). The results showed that neither the participants’ inclination for conscious movement investment, nor the direction of conscious attention affected inhibition performance. In Experiment 2, participants performed the stop-signal task, which is more attention demanding than the go/no-go task. The results showed that participants with a high or low inclination for conscious movement investment did not differ in inhibition performance. In Experiment 3 an ego-depletion procedure was included that limits resources for conscious movement investment. Before and after this ego-depletion procedure, participants performed the stop-signal task. The results showed that participants with a high inclination for conscious movement investment slowed down inhibition when they felt mentally depleted, while no slowing down of inhibition was found among participants who felt less depleted and/or had a low inclination for conscious movement investment. Together, the study provides evidence that increased conscious movement investment is beneficial for movement inhibition. Yet, these effects only emerge against the dynamic background of interacting individual (e.g., inclination for conscious movement investment, available attentional resources) and task constraints (e.g., task difficulty).

## 1 Introduction

Movements can be executed consciously or automatically. In the current study, we are interested in the conscious monitoring and control of the movement or conscious movement investment. We define conscious movement investment^1^ as performer’s conscious or selective attention to movement in order to monitor and/or control how it unfolds, which is accompanied by a conscious use of some degree of explicit movement-related knowledge in the ongoing movement. There is broad consensus that, for well-learned motor skills, conscious movement investment disrupts movement automaticity, leading to impaired movement performance (Wulf and Prinz, 2001; Beilock et al., 2002; Gray, 2004, 2006; Ford et al., 2005; Beilock and Gray, 2012). For example, skilled soccer players slowed down dribbling with their dominant foot when asked to consciously attend to how they touched the ball; similarly, experienced golfers showed larger aiming errors (i.e., the distance from the centre of the target) when triggered to pay attention to the follow-through motion of the clubhead (Beilock et al., 2002). Such conscious movement investment is also thought to underlie breakdown of performance in high-pressure situations (Masters, 1992). Intriguingly, however, conscious movement investment may not necessarily adversely affect the execution of movements: intentionally stopping or inhibiting movements may be facilitated by conscious movement investment (Beilock and Gray, 2012; Park et al., 2020). The purpose of the current research is to further explore this putative relationship by examining whether variations in the inclination for and/or actual degree of conscious movement investment affects inhibition.

Unlike many studies investigating how conscious movement investment affects movement outcomes, there have been only two studies so far assessing its relationship with movement inhibition (Beilock and Gray, 2012; Park et al., 2020). Beilock and Gray (2012), compared inhibition of a golf putting stroke in novices and high-skilled golfers. They assumed that novices would naturally show higher levels of conscious movement investment compared to high-skilled golfers who would show much more automatic monitoring and control of movement. This contention is based on the theory of skill acquisition (Fitts and Posner, 1967), which posits that, during the initial stage of learning, movement execution is controlled by unintegrated control structures which are held in working memory. As learning progresses, performers develop encapsulated procedural structures that allow for the automatic, nonconscious monitoring and control of movement. In the study of Beilock and Gray (2012), they asked participants to putt on an indoor green. A stop signal (i.e., an auditory tone) appeared at the backswing or downswing of strokes on some trials (i.e., 33%). Then participants were required to halt the putting stroke as quickly as possible after hearing a stop signal. They found that the novices were faster in inhibiting the stroke than the high-skilled golfers, especially when the stop signal was present during the downswing. Beilock and Gray (2012) argued that novices were faster in stopping the stroke, because they were already consciously investing (i.e., monitoring and/or controlling) to the putting movement, while high-skilled golfers first had to shift attention toward the movements (Gray, 2009; Beilock and Gray, 2012).

Recent observations by Park et al. (2020) were (partially) consistent with this result. While Beilock and Gray (2012) assumed that their participants, depending on skill level, invested different degrees of consciousness in movements, Park et al. (2020) tried to more directly pinpoint the relationship between conscious movement investment and inhibition. In fact, they reasoned that inhibition would allow performers to control the degree of conscious movement investment. They thus expected that movement inhibition would negatively correlate with performers’ inclination to consciously invest in movement monitoring and control.^2^ To this end, Park et al. (2020) measured participants’ inclination for conscious movement investment via a questionnaire. The questionnaire, referred to as the Movement-Specific Reinvestment Scale (MSRS), was originally introduced and validated by Masters et al. (1993), see also Kal et al., 2016) to assess performers’ inclination for conscious (re-)investment in high-pressure situations. The MSRS consists of 10 statements about moving in general that gauge movement self-consciousness and conscious movement processing. Previous studies using the MSRS have shown that performers with a high MSRS score more likely demonstrate high levels of investment of explicit movement-related knowledge in motor performance and learning (Malhotra et al., 2015; van Ginneken et al., 2017, 2018) and increased likelihood of performance breakdown in high-pressure situations due to de-automatisation movement monitoring and control (Maxwell et al., 2006).

Park et al. (2020) asked participants to complete the MSRS and the go/no-go task (i.e., GNG task). The GNG task is one of the classic tasks for measuring movement inhibition, requiring participants to respond to go signals (i.e., go trials) or withhold a response in response to stop signals (i.e., no-go trials or stop trials). The frequency of commission errors (i.e., the probability of executing a response on no-go trials) is the primary inhibition measure in the GNG task (Verbruggen and Logan, 2008). Park et al. (2020) did not find a correlation between the MSRS score and the frequency of commission errors. However, they did reveal a negative correlation between the MSRS score and the variability in the go reaction time. The variability in the go reaction time has been suggested to be positively correlated with the frequency of commission errors (Bezdjian et al., 2009). Hence, if anything, this observation suggests that individuals with a high inclination for conscious movement investment show better movement inhibition. This opposed Park et al.’s (2020) original expectation but is consistent with Beilock and Gray (2012) hypothesis.

The empirical evidence for a relationship between conscious movement investment and movement inhibition is still weak. Firstly, Park et al. (2020) only provided evidence for such a relationship for the variability in the go reaction time, but not for the frequency of commission errors, which is the primary index for movement inhibition for the GNG task (Verbruggen and Logan, 2008). Secondly, in the GNG task there is a consistent signal-response mapping, that is, go and no-go signals are mutually exclusive (Verbruggen and Logan, 2008). The consistent mapping between the no-go signal and no-go response allows an automatic triggering of the no-go response and requires relatively low levels of conscious attention on the task (Logan and Cowan, 1984; Verbruggen and Logan, 2008). If conscious attention to the task is low, then attention to the movement is likely low as well. Thus, the GNG task may require insufficient conscious movement investment, even for participants with a high inclination for conscious movement investment. Thirdly and relatedly, Beilock and Gray (2012) and Park et al. (2020) did not directly manipulate conscious movement investment but capitalised on individual differences in the likelihood that participants would consciously invest in the tasks. Hence, it remains unknown to what degree participants were actually consciously attending when performing the GNG task or golf task. It is therefore important to further explore the relationship between the (inclination for) conscious movement investment and movement inhibition under conditions that require varying amounts of conscious movement investment.

Accordingly, to further explore the relationship between the (inclination for) conscious movement investment and movement inhibition, three experiments were conducted. In Experiment 1, we directly manipulated conscious movement investment and investigated whether increased and reduced conscious movement investment differently affected movement inhibition as assessed by the GNG task. A common approach to manipulate conscious movement investment is to provide participants with instructions that encourage them on where to focus (Wulf, 2013). It has been shown that focusing on the movements themselves (i.e., internal focus) increases conscious investment of explicit movement-related knowledge while focusing on the effects of movements (i.e., external focus) reduces conscious movement investment (Poolton et al., 2006). Therefore, to manipulate conscious movement investment, internal and external foci of attention were utilized. We also tested to what degree the effect of conscious movement investment on inhibition was moderated by the participants’ inclination for conscious movement investment as assessed by the MSRS.

In Experiment 2, rather than using the GNG task, we used the stop-signal task (SST) to assess movement inhibition. The SST requires participants to perform a choice reaction time task (CRTT) by pressing buttons in response to different go signals. The stop signal (e.g., a change in the colour of the go signal) occurs shortly after the go signal, requiring participants to withhold the response (Verbruggen and Logan, 2008). Because the stop response is preceded by a combined go and stop signal, the mapping between the stop signal and stop response is inconsistent. This inconsistency necessitates that participants continually prepare to stop, thereby requiring higher levels of conscious attention in the task compared to the GNG task (Verbruggen and Logan, 2008), presumably increasing conscious movement investment as well. Hence, in Experiment 2, we examined the relationship between the inclination for conscious movement investment, as measured with the MSRS, and movement inhibition on the SST.

Finally, in Experiment 3, we manipulated the capacity for conscious movement investment by depleting participants’ resources upon which conscious movement investment depends – that is, the resources for self-control. Self-control refers to the capacity to override or alter predominant responses, and it represents a limited cognitive resource that depletes with exertion. The state that follows the depletion of self-control is referred to as ego-depletion (Baumeister et al., 2007; Hagger et al., 2010). In the SST, participants presumably invest more conscious effort in task execution compared to the GNG task, especially individuals with higher inclination for conscious movement investment. Maintaining conscious movement investment requires self-control resources as it is one form of self-control (Bruya, 2010). Thus, in Experiment 3, we manipulated the capacity for conscious movement investment using a validated dual-task paradigm for ego-depletion (Hagger et al., 2010; Pohl et al., 2013). We assessed whether reducing the capacity for conscious movement investment influenced movement inhibition as assessed by the SST. Like in Experiment 1, we also tested whether the effects, if any, were moderated by the inclination for conscious movement investment.

## 2 Experiment 1

A few studies have suggested that a positive association exists between conscious movement investment and movement inhibition (Beilock and Gray, 2012; Park et al., 2020). This evidence is derived from a comparison across groups of individuals, who presumably showed different degrees of conscious investment in movement monitoring and control. However, the degree of conscious movement investment was not directly manipulated in those studies. Hence, in Experiment 1, we examined the effect of various degree of conscious movement investment on inhibition in the GNG task. Additionally, following Park et al. (2020), we investigated whether the inclination for conscious movement investment moderated this effect. To increase conscious movement investment, we instructed participants to direct attention to the movement while performing the GNG task (i.e., internal focus; Wulf and Su, 2007) and compared this with their performance when instructed to focus on the effect of the movement (i.e., external focus). It is presumed that an internal focus increases conscious attention to the movement, which not only results in less fluent (or automatic) movement execution (Kal et al., 2013) but also in worse performance outcomes compared to an external focus (Wulf et al., 1998; Wulf and Prinz, 2001; Wulf and Su, 2007; Wulf, 2013).

Hence, in Experiment 1, we aimed to manipulate participants’ levels of conscious movement investment in the GNG task by requiring them to perform under internal and external focus instructions and measured their inclination for conscious movement investment via the MSRS. We predicted that internal focus instructions and the concomitant increase in conscious movement investment would result in increased inhibition as reflected by a lower frequency of commission errors in comparison to external focus instructions, especially among the participants who show a strong inclination for conscious movement investment.

### 2.1 Materials and methods

#### 2.1.1 Participants

A total of 41 participants (age: 31.0 ± 3.8 years old, 16 males, 25 females) from Taiwan and the Netherlands were recruited through the internet. G*power (version 3.1.9.6) showed at least 34 participants were need for detecting a within-between interaction with a power level of 80%, a moderate effect size of 0.25 at a significance level of 0.05. Participants self-reported handedness: 37 were right-handed, 3 were left-handed, and 1 was ambidexter. None of the participants reported a history of neurological or motor problems, or of having visual impairments. The study was approved by the local institution’s ethics committee and participants gave informed consent before the start of the experiment.

#### 2.1.2 Tasks and materials

##### 2.1.2.1 MSRS

Movement-Specific Reinvestment Scale comprises of 10 items loading on 2 factors (Masters et al., 2005). Five items assess conscious movement processing, such as, “I try to figure out why my actions failed,” while the other five items gauge, according to a recent interpretation, conscious movement monitoring (Malhotra et al., 2015), for example, “I am aware of the way my body works when I am carrying out a movement.” Each item is rated on a 6-point Likert scale, ranging from *1 (strongly disagree) to 6 (strongly agree).* The cumulative scores range from 10 to 60, the higher the score the stronger the inclination for conscious movement investment.

##### 2.1.2.2 GNG task

**FIGURE 1:**
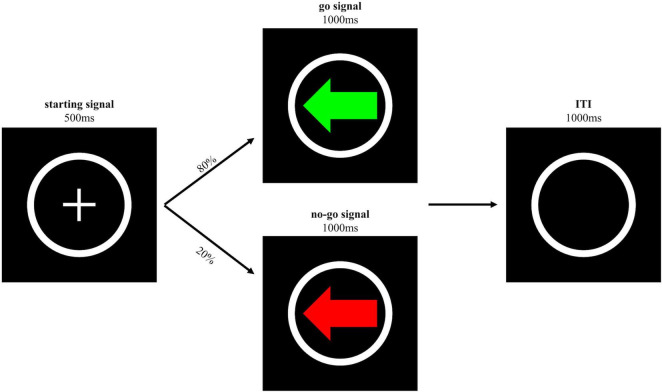
Schematic representation of the trial sequence of the GNG task. The go and no-go signal were either a left-pointing or right-pointing arrow, only the left-pointing arrow is shown here.

The GNG task (see Figure 1) comprised of an initial practice block of 5 trials and two test blocks of 50 trials with go and no-go trials. In each block, the ratio of go trials and no-go trials was 4:1 (Park et al., 2020). On each trial, a white cross surrounded by a white circle (i.e., the starting signal) was displayed at the centre of the screen for 500 ms indicating the beginning of the trial, which was followed with a right-pointing or left-pointing arrow displayed for 1,000 ms. On go trials, a green left-pointing or right-pointing arrow served as the go signal. Participants were asked to press the S or K key as quickly as possible after the green left-pointing or right-pointing arrow appeared on the screen, respectively. On no-go trials, a red left-pointing or right-pointing arrow served as the no-go signal and participants were instructed not to press any key if a red signal appeared. Both go signals and no-go signals remained visible until participants pressed one of the keys or after 1,000 ms had passed. To increase conscious investment in the movement, participants were required to use their left and right ring fingers instead of their index fingers as is normally required in the GNG task. On go trials, if participants had not responded within 1,000 ms or if they had pressed the wrong key within 1,000 ms, the trial was considered as an omission trial or a wrong trial, and feedback was given, stating “You should have pressed” or “Wrong key” for 1,250 ms, respectively. On no-go trials, if participants pressed any key within 1,000 ms, the trial was considered as a commission trial and feedback was provided, stating “You should not have pressed” for 1,250 ms. A black screen with a white circle frame was shown for 1,000 ms after a go or no-go signal, or the feedback disappeared.

#### 2.1.3 Procedure

Because of the COVID-19 pandemic, the entire experiment, including the MSRS and the GNG task, was programmed on the online website PsyToolKit (Stoet, 2010, 2017). PsyToolKit has been shown to be comparable to E-prime 3.0, a widely used psychological research tool in laboratory setting (Kim et al., 2019). Besides, the official website states that the internet speed does not influence the response times (PsyToolkit, 2021). Participants were asked to use a laptop or a desktop computer and a keyboard in a quiet room. They were asked to sit comfortably in front of the screen and first completed the MSRS. Following that, they performed the GNG task in the internal focus and the external focus conditions, the order of which was counterbalanced among participants. There was short break between blocks and two conditions.

The internal and external focus conditions only differed in how the instructions were formulated. In the internal focus condition, the participants were instructed to “use the left ring finger for pressing the S key and the right ring finger for pressing the K key. In doing so, focus your attention to your *finger movement*.” In the external focus condition, they were instructed to “use the left ring finger for pressing the S key and the right ring finger for pressing the K key. In doing so, focus your attention to the motion of the *key*.” To reinforce conscious movement investment throughout the GNG task, a cue with the text “keep your attention on your finger movement” or “keep your attention on the key motion” showed up after the 5th, 10th, 20th, 30th, and 40th trial of two test blocks in both the internal and external focus condition.

The participants performed the GNG task after they had participated in the SST^3^ to be reported in Experiment 2.

#### 2.1.4 Data analysis

##### 2.1.4.1 Dependent variables

The MSRS score was calculated for each participant. For the two conditions, the mean reaction time for correct go responses (i.e., Go_Correct_RT), the ratio of correct responses to all responses on go trials (i.e., Go_Correct_Rate), the ratio of commission errors to all responses on no-go trials (i.e., No-Go_Commission_Rate) and the variability in reaction time on go trials (i.e., Go RTV; the ratio of the SD to the mean reaction time) were determined. The abbreviations of dependent variables are displayed in the Appendix.

##### 2.1.4.2 Statistical analysis

We used R Studio (Version 1.4.1106) for data pre-processing and SPSS (Version 26) for statistical analysis. To categorize participants, we applied a mean split to the MSRS score, creating two groups: high MSRS and low MSRS. Next, the Go_Correct_RT, Go_Correct_Rate, No-Go_Commission_Rate, and Go RTV were submitted to separate 2 (group: high MSRS, low MSRS) by 2 (condition: internal, external) ANOVAs with repeated measures on the last factor. The simple main effect analysis was planned to follow up significant effects. Effect sizes were calculated with partial eta-squared (i.e., $\eta_{p}^{2}$). Values of 0.01, 0.06, and 0.14 were interpreted as small, medium, and large, respectively.

### 2.2 Results

According to the mean MSRS score (*M* = 41.76, SD = 8.97), 22 participants were assigned to the high MSRS group (*M* = 48.45, SD = 3.66), and 19 participants to the low MSRS group (*M* = 34.00, SD = 6.69). Table 1 shows that no differences between groups and conditions were apparent. Accordingly, the ANOVA did not reveal significant effects for group, condition, and group by condition (Table 2).

**TABLE 1:** Descriptives of Experiment 1.

	High MSRS		Low MSRS	
	Internal	External	Internal	External
Go_Correct_RT (ms)	393.76 (37.80)	397.73 (39.58)	401.62 (34.80)	405.00 (55.62)
Go_Correct_Rate (%)	98.69 (1.42)	98.58 (2.32)	98.82 (1.89)	99.41 (0.87)
No-Go_Commission_Rate (%)	3.18 (5.47)	2.05 (4.80)	1.84 (2.99)	2.63 (4.82)
Go RTV	0.13 (0.04)	0.14 (0.05)	0.13 (0.06)	0.13 (0.04)

The number in parenthesis indicate SD.

**TABLE 2:** Statistics of Experiment 1.

	Group			Condition			Group × condition		
	*F*(1,39)	*p*	* $\eta_{p}^{2}$ *	*F*(1,39)	*p*	* $\eta_{p}^{2}$ *	*F*(1,39)	*p*	* $\eta_{p}^{2}$ *
Go_Correct_RT (ms)	0.47	0.50	0.01	0.25	0.62	0.01	<0.01	0.97	<0.01
Go_Correct_Rate (%)	1.40	0.24	0.04	0.43	0.51	0.01	0.94	0.34	0.02
No-Go_Commission_Rate (%)	0.09	0.77	<0.01	0.06	0.82	<0.01	1.72	0.20	0.04
Go RTV	0.43	0.52	0.01	0.02	0.89	<0.01	0.77	0.39	0.02

### 2.3 Discussion

In Experiment 1, we investigated whether the degree of conscious movement investment affects inhibition. To manipulate conscious movement investment, participants performed the GNG task with internal and external focus instructions. We hypothesized that an internal focus of attention increases conscious movement investment and would result in enhanced inhibition performance compared to an external focus of attention, especially for participants with a high inclination for conscious movement investment.

Neither participants’ inclination for conscious movement investment nor the momentary degree of conscious attention for movement execution influenced inhibition performance in the GNG task. That is, the most critical indicator, commission rate, did not differ as a function of group or focus condition. Also, the more indirect measures for inhibition performance did not show any differences (see Table 1). This included the variability in reaction time of go trials (i.e., Go RTV), which was previously reported to correlate to the MSRS score (Park et al., 2020). These findings suggest that inhibition was not influenced by the degree of conscious movement investment or the inclination to consciously invest in movement execution.

One concern with the generality of this conclusion is that our manipulation of focus of attention may have been insufficiently successful. Typically, it is found that increased attention to the movement disrupts performance, especially if they concern well-developed automatic movements, such as the current button-press movements (Wulf and Su, 2007; Kal et al., 2013). For the current experiment this would have meant decreased performance for the go response in the internal focus condition. However, the correct rate and reaction time on go trials did not differ significantly between two focus of attention instructions. This accords with Ziv and Lidor (2021), who recently reported that they failed to show a difference between internal and external focus of attention instructions on a CRTT and a Simon task. Perhaps the simple button-press movements are immune to the attentional focus manipulation and/or only demand low levels of conscious movement investment. However, this remains an assumption, as Experiment 1 did not evaluate how well the two groups followed the focus of attention instructions. Future research should address this by assessing it directly. For instance, a post-manipulation questionnaire could be an effective method (Lawrence et al., 2011). An additional alternative explanation for the observed lack of difference is that participants may have learned the mapping between no-go signal and stop response, potentially reducing any contrast in conscious movement investment across the two attentional conditions. Indeed, Verbruggen and Logan (2008) previously found that inhibition became quickly automatic in the GNG task. Hence, it is important to reconsider the relationship between conscious movement investment and inhibition with task constraints that potentially induce higher levels of conscious movement investment.

## 3 Experiment 2

In Experiment 2, we investigated the purported positive relationship between conscious movement investment and movement inhibition using the SST, which is argued to require increased levels of conscious attention to the task compared to the GNG task (Verbruggen and Logan, 2008). In the SST, the mapping between a no-go or stop signal and a stop response varies, in contrast to the consistent mapping between a no-go signal and a stop response in the GNG task (Verbruggen and Logan, 2008). The inconsistent mapping in the SST means that participants continuously need to be prepared to inhibit a go response. In this sense, participants must pay greater conscious attention to the SST, which is presumably associated with greater conscious investment in movement. Another advantage of using the SST is that it provides a more quantitative measure for movement inhibition, instead of only estimating the frequency of commission errors. That is, the primary measure is the stop-signal reaction time (SSRT), indicating the time needed to halt an intended movement response. Consequently, in Experiment 2, we determined the SSRT and examined whether it differed between participants who show high and low inclinations to conscious movement investment, using the MSRS. We hypothesized that the SSRT would be shorter for participants with a high inclination for conscious movement investment compared to those with a low inclination for conscious movement investment.

### 3.1 Materials and methods

#### 3.1.1 Participants

The participants were the same as the Experiment 1.

#### 3.1.2 Tasks and materials

##### 3.1.2.1 MSRS

See Experiment 1.

##### 3.1.2.2 The choice reaction time task

**FIGURE 2:**
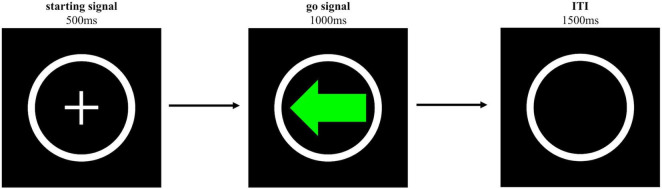
Schematic representation of the trial sequence of the CRTT. The go signal was either a green left- or right-pointing arrow. Only the left-pointing arrow is shown here.

Participants performed a CRTT (see Figure 2). The CRTT consisted of one practice block of 16 trials and 1 test block of 48 trials. On each trial, a white cross surrounded by a white circle (i.e., starting signal) was displayed at the centre of the screen for 500 ms, indicating the beginning of the trial. This was followed by a green left- or right-pointing arrow, with a 1:1 ratio, that served as the go signal and was displayed for 1,000 ms. Participants were asked to press the A key with the left index finger or the L key with the right index finger as quickly as possible, when the left- or right-pointing arrow was shown, respectively. The go signals remained on the screen until participants pressed one of the keys or when 1,000 ms had passed. On each trial, if participants did not respond within 1,000 ms or pressed the wrong key within 1,000 ms, the trial was considered an omission trial or a wrong trial, and feedback was provided stating “You should have pressed” or “Wrong key” for 1,250 and 2,000 ms, respectively. If reaction time was longer than 500 ms (on correct go trials), feedback was provided stating “Too slow” for 1,250 ms to re-emphasize being as quickly as possible. A black screen with a white circle frame was shown for 1,500 ms after the go signals or after the feedback disappeared.

##### 3.1.2.3 SST

**FIGURE 3:**
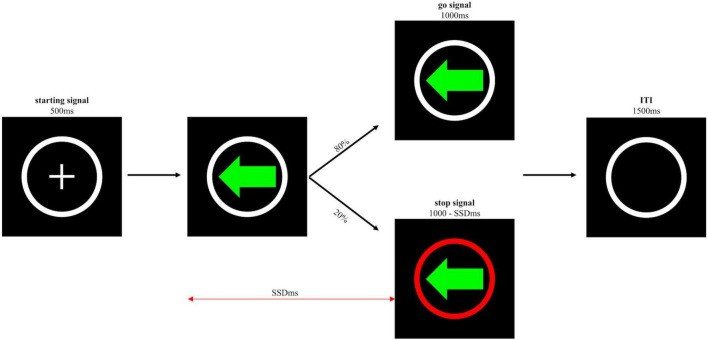
Schematic representation of the trial sequence of the SST. The go or stop signal was either a left or a right-pointing arrow. Only the left-pointing arrow is shown here.

The SST (see Figure 3) comprised of one practice block of 20 trials and four test blocks of 60 trials. The ratio of go trials and stop trials was 4:1 in each block. The go trials in the SST were identical to the trials in the CRTT. In other words, the required response to go signals and feedback were identical. Participants were asked to respond as quickly as possible. Like the go trials, the same green left- and right-pointing arrows surrounded by a white circle were shown (with a ratio of 1:1), but with the white circle turning red on stop trials. This change in colour served as the stop signal, occurring after the go signal with varying time delays known as the stop-signal delay (SSD). The longer the SSD, the more difficult it is to inhibit the go response. The SSD was varied using the tracking procedure (Verbruggen et al., 2019). More specifically, when participants successfully withheld a response to a go signal, the SSD increased by 50 ms on the next stop trial; when participants failed to inhibit the go response, the SSD decreased by 50 ms on the next stop trial. The initial SSD was set 183 ms, and the minimum SSD was set 33 ms. The go and stop signals remained on the screen until participants pressed one of the keys or when 1,000 ms had passed. If participants failed to inhibit the response on stop trials, feedback was given stating “You should not have pressed” for 1,250 ms. A black screen with a white circle frame was shown for 1,500 ms after the go or stop signals or after the feedback disappeared.

#### 3.1.3 Procedure

The experiment was done online. Participants were asked to use a laptop or a desktop computer and a keyboard in a quiet room and asked to sit comfortably in front of the screen. They first completed the MSRS. Following that, they performed the CRTT and SST in this sequence. There was short break between blocks and tasks.

#### 3.1.4 Data analysis

##### 3.1.4.1 Dependent variables

The MSRS score was calculated for each participant. For the CRTT, we determined the following dependent variables: the mean reaction time on all go trials (i.e., CRTT_Go_RT) and the ratio of correct responses to all responses (i.e., CRTT_Correct_Rate). For the SST, we determined the mean reaction time on all go trials (SST_Go_RT), the ratio of correct responses to all responses on go trials (i.e., SST_Go_Correct_Rate), the mean reaction time on stop trials where participants failed to stop or cancel the response (i.e., SST_Uncancelled_RT), the ratio of uncancelled responses to all responses on stop trials (i.e., SST_Uncancelled_Rate), and the mean SSD (i.e., SST_SSD). Because slowing down the go response might distort the estimation of SSRT (Band et al., 2003; Boehler et al., 2012; Verbruggen et al., 2013), response slowing was measured by subtracting the CRTT_Go_RT from the SST_Go_RT to check for the presence of distortion in each participant. Finally, and most importantly, the SSRT was estimated. The SSRT is the time interval between the onset of the stop signal and the end of the stop process. This cannot be measured directly and is therefore estimated based on the independent horse-race model (Verbruggen et al., 2019). This model assumes that movement inhibition is a race between two independent processes: the go process triggered by the go signal and the stop process triggered by the stop signal. If the go process is faster than the stop process, the response cannot be inhibited. However, if the stop process is faster than the go process, the response will be inhibited. We used the integration method to estimate the SSRT (Matzke et al., 2018). The moment at which the stop process finishes is estimated by integrating the distribution of reaction times on go trials and identifying the point at which the integral equals the SST_Uncancelled_Rate. In other words, the end of the stop process corresponds to the reaction time on go trials at the percentile that corresponds to the SST_Uncancelled_Rate (*n*-th RT) (i.e., including go trials with a wrong response or an omission response, where an omission response was replaced by the maximum reaction time, that is, 1,000 ms). For example, if the total number of go trials is 300, and the SST_Uncancelled_Rate is 0.45, then the *n*-th RT is the 135th fastest RT on Go trials (i.e., 300 × 0.45). SSRT is then estimated by subtracting SST_SSD from the *n*-th RT (i.e., SSRT = *n*-th RT – SST_SSD). In this study, we adopted the block-wised integration method to estimate the SSRT. Specifically, SSRT in each block was estimated solely, and then the summary SSRT was calculated by averaging the SSRTs of the four blocks (Verbruggen et al., 2013; Matzke et al., 2018). The abbreviations of dependent variables are displayed in the Appendix.

##### 3.1.4.2 Statistical analysis

R studio (Version 1.4.1106) was used to pre-process the data, and SPSS (Version 26) was used for statistical analysis. We used a mean split for the MSRS score to assign individual participants to either the high MSRS group or the low MSRS group. Next, to reliably estimate the SSRT, several assumptions were tested based on the independent horse-race model (Logan and Cowan, 1984). First, if for an individual participant the SST_Uncancelled_RT was longer than the SST_Go_RT, then the participant was excluded because this would violate the assumption of independence of the go and stop processes that underpins the horse-race model (Verbruggen et al., 2019). However, no participants were excluded for this reason. Second, if a participant’s SST_Uncancelled_Rate was higher than 85% or lower than 15%, the participant would also be excluded (Chen et al., 2009). Again, this did not apply to any participant. Finally, the independent *t*-tests were performed to examine differences between the high and low MSRS group. Significant levels were set at the 0.05 level. Cohen’s *d* was calculated to estimate the effect size with values of 0.2, 0.5, and 0.8 considered as small, medium, and large, respectively.

### 3.2 Results

The assignment of participants to the high and low MSRS groups was identical as in Experiment 1. Table 3 reports the results of Experiment 2. We did not find any difference between the two groups. Importantly, there were no significant differences between the two groups in SSRT, *t*(39) = −1.63, *p* = 0.11, *d* = −0.51.

**TABLE 3:** Descriptives and statistics of Experiment 2.

	High MSRS	Low MSRS	*t*(39)	*p*	*d*
SST_Go_RT (ms)	422.21 (43.21)	425.31 (26.59)	0.27	0.79	0.09
SST_Go_Correct_Rate (%)	98.32 (1.94)	99.23 (0.91)	1.88	0.07	0.59
SST_Uncancelled_Rate (%)	54.07 (5.94)	51.32 (2.22)	−1.91	0.06	−0.60
Response slowing (ms)	51.73 (27.31)	52.71 (25.26)	0.12	0.91	0.04
SSRT (ms)	277.01 (41.98)	257.92 (30.97)	−1.63	0.11	−0.51

The number in parenthesis indicate SD.

### 3.3 Discussion

In Experiment 2, we compared inhibition performance on the SST for participants with a high and low inclination for conscious movement investment. Unlike the GNG task used in Experiment 1, the inconsistent signal-response mapping in the SST would increase the level of conscious involvement in task, likely increasing conscious movement investment as well. We hypothesized that individuals with a high inclination for conscious movement investment would need less time to stop in response to the stop signal (i.e., SSRT) compared to individuals with a low inclination for conscious movement investment.

However, the SSRT, which is considered the main indicator for inhibition performance on the SST, did not differ between the participants with a high and low inclination for conscious movement investment. As per Experiment 1, it is possible that simple button-press movement responses may not demand a high level of conscious movement investment, even with inconsistent mapping between signal and response (Ziv and Lidor, 2021). Nonetheless, participants with a high inclination for conscious movement investment showed a nonsignificant tendency to more often forego a go response (i.e., reflecting a lower accuracy rate on go trials) and to fail to inhibit after a stop signal in comparison to participants with a low inclination for conscious movement investment (see Table 3). Possibly, the participants with high inclination for conscious movement investment might have differently traded off the requirements for a fast response and successful inhibition (Castro-Meneses et al., 2015; Schmitt et al., 2018). However, with the current smaller sample size, caution must be applied, also in interpreting the null finding for the more critical SSRT.

In sum, we found no support in the current experiment for the proposal that participants with a high propensity for conscious movement investment would show shorter inhibition times. Yet, with the small sample size, this finding needs to be reassessed. In that respect, one might anticipate that if, in addition to differences in participants’ inclination for conscious movement investment, the capacity or resources for actual conscious investment in movement were manipulated, this would provide a stronger test of the putative influence of conscious movement investment on inhibition.

## 4 Experiment 3

In Experiment 3, we manipulated the capacity for conscious movement investment by depleting the resources for self-control. According to the self-regulation strength model, the resources for acts of self-control are limited (Baumeister et al., 2007). Due to this limited capacity, if self-control resources are depleted (i.e., the energy for mental activities is low), then self-control will be impaired in a subsequent task (Baumeister et al., 2007). Consequently, in Experiment 3, participants first performed the SST to determine baseline inhibition performance. The self-control resources of the participants were then depleted using a transcriptional task (Englert and Bertrams, 2014), after which they performed the SST again. This allowed us to test whether a reduced capacity for conscious movement investment following the ego-depletion resulted in prolonged SSRT, and whether this would be moderated by the participant’s inclination for conscious movement investment. We hypothesized that participants with a high inclination for conscious movement investment would show larger increase in SSRT after depletion (i.e., in the post-intervention test compared to the baseline test) than participants with a low inclination for conscious movement investment.

### 4.1 Materials and methods

#### 4.1.1 Participants

A total of 40 participants (age: 24.3 ± 3.4 years old, 16 males, 24 females) were recruited through the internet, who were mostly from Indonesia, the Netherlands, and France. They did not participate in Experiments 1 and 2. A medium-to-large effect for ego-depletion on self-control dependent variables was reported, that is, Cohen’s *d* = 0.62 (Hagger et al., 2010). We converted, Cohen’s *d* in f based on the equation $\frac{d}{2}$, resulting in a f of 0.31(Correll et al., 2020). G*Power (version 3.1.9.6) showed that at least 36 participants were needed to detect a three-way interaction with a power level of 80% at a significance level of 0.05. None of the participants reported a history of neurological or motor problems or visual impairments. The experiment was approved by the local institution’s ethics committee and participants gave their informed consent before starting the experiment.

#### 4.1.2 Tasks and materials

##### 4.1.2.1 MSRS

See Experiment 1.

##### 4.1.2.2 CRTT

The CRTT comprised of one practice block of 40 trials. However, participants could quit the practice block after having pressed the correct key in the first 20 trials. This was followed by a test block of 50 trials. On each trial, a white cross (i.e., starting signal) was displayed at the centre of the screen for 500 ms, indicating the beginning of the trial, followed by a black screen that was presented for 200 ms. Next, a white circle appeared on the left or right side of the screen for 1,000 ms. This white circle served as the go signal. Participants were instructed to press the F key with the left index finger or the J key with the right index finger as quickly and accurately as possible when the white circle appeared on the left or right side of the screen, respectively. If participants pressed the wrong key within 1,000 ms, the trial was considered a wrong trial and feedback was given stating “Wrong key.” The feedback was shown for 2,000 ms. The go signal remained on the screen until participants pressed one of the keys or after 1,000 ms had passed. A black screen was shown for 1,000 ms after a go signal or the feedback had disappeared.

##### 4.1.2.3 SST

The SST^4^ procedure adopted in Experiment 3 was based on previous research (Chen et al., 2009; Wang et al., 2013) and differed from the SST used in Experiment 2. It consisted of two phases; the first phase was to measure a participant’s SSD at which the uncancelled rate was around 50% (i.e., the critical SSD), the second phase was to measure a participant’s SSRT. This SST procedure helped to reduce the number of trials in the second phase compared to Experiment 2 (Chen et al., 2009). The SST had identical go trials as the go trials in the CRTT; that is, a white circle appeared at the left or right side of the screen. On stop trials, a second white circle, which is the stop signal, appeared at the centre of the screen following the go signal. Participants were asked not to press any key if it appeared. Both the go and stop signal remained on the screen until participants pressed one of the keys or 1,000 ms had passed. On go trials, the trial was considered a wrong trial if participants pressed the wrong button within 1,000 ms, and feedback was given stating “Wrong key.” The trial was considered an omission trial if participants did not press any button within 1,000 ms. If they pressed the correct key, but the reaction time was longer than 500 ms, feedback was given stating “Response too slowly.” This was done to prevent participants from slowing down the go response. On stop trials, if participants pressed any key within 1,000 ms, the trial was considered a commission trial, and feedback was given stating “You should not have pressed.” All feedback statements were shown for 2,000 ms. A black screen was displayed for 1,000 ms after a go or stop signal, or after the feedback had disappeared.

The SST consisted of two phases. *Phase 1*: In this first phase, the critical SSD was determined. It consisted of a practice block of 32 trials and several test blocks of 32 trials. The go and stop trials, with a ratio of 3:1 in each block, were presented in random order. For each participant, we manipulated the SSD across blocks to obtain the critical SSD. The SSD was set at 183 ms in the first block. If the cancelled rate was higher than 63%, the SSD increased by 50 ms in the next block; if the cancelled rate was lower than 38%, the SSD decreased by 50 ms in the next block; and if the cancelled rate was between 63% and 38%, the SSD remained the same in the next block. The first phase ended once the participant’s cancelation rate was between 63% and 38% in two consecutive blocks. The SSD in these blocks was considered as the critical SSD. *Phase 2*: The second phase consisted of a practice block of 24 trials and three test blocks of 48 trials. The ratio of go trials and stop trials was 3:1 in each block, and the types of trials were presented randomly. In each block, there were three different SSDs: (1) critical SSD − 50 ms; (2) critical SSD; and (3) critical SSD + 50 ms. Each SSD appeared four times in each test block in random order.

##### 4.1.2.4 Transcriptional task

The Transcriptional Task was used to induce ego-depletion. The Transcriptional Task has been applied and validated in previous studies (Wolff et al., 2013; Englert and Bertrams, 2014). Following Englert and Bertrams (2014), participants received a neutral text (“Stalagmites and Stalactites,” 2011) and were asked to copy it. Participants in the ego-depletion group (i.e., EDG) were asked to copy the text, but requested to omit letters “a” and “i” so that the word “stalagmite” would then be typed as “stlgmte” and “stalactites” would be “stlcttes.” Because participants in this group needed to intentionally override the writing habits when they transcribed the text, self-control strength was expected to get depleted (Englert and Bertrams, 2014). Participants in the control group (i.e., CG) were instructed to copy the text verbatim, which did not require self-control strength. To verify (perceived) depletion, after participants had finished the Transcriptional Task, participants completed three questions on a 4-point Likert scale from *not at all* (1) to *very much* (4) (Bertrams et al., 2010). These included, “How difficult did you find the task?”, “How depleted do you feel at the moment?”, and “How effortful did you find the task?”). The total score was defined as the depletion scores.

#### 4.1.3 Procedure

**FIGURE 4:**
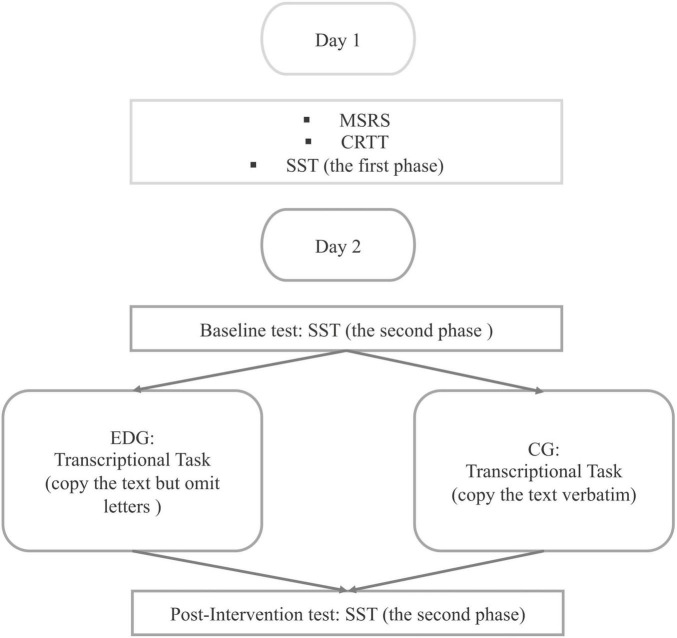
The design of Experiment 3.

The experiment was done online. Figure 4 shows the design of Experiment 3. Participants were randomly assigned to either, the EDG and CG. Data collection took 2 days for each participant. On the first day, participants first completed the MSRS, and then performed the CRTT followed by phase 1 of the SST. At the second day, phase 2 of the SST was performed twice. After, the first or baseline test was completed, they performed the Transcriptional Task and then performed phase 2 of the SST again, the second or post-intervention test. After each block and task, participants were granted a short break, however, they were asked to perform the post-intervention test immediately after they had completed the Transcriptional Task (i.e., without a break).

#### 4.1.4 Data analysis

##### 4.1.4.1 Dependent variables

The total score of the MSRS was calculated (i.e., MSRS score). For the CRTT, we measured the CRTT_Go_RT. For the SST, SST_Go_RT, SST_Go_Correct_Rate, SST_Uncancelled_RT, SST_Uncancelled_Rate, and most importantly, the SSRT were measured separately for the baseline and post-intervention test. The abbreviations of dependent variables are displayed in the Appendix. The SSRT for each of three SSDs was calculated using the integration method (including go trials with a wrong response or an omission response, where an omission response was replaced by the maximum reaction time, that is, 1,000 ms) and then averaged to obtain the mean SSRT (Wang et al., 2013). For the calculation of the SSRT of each SSD, SST_Uncancelled_Rates of 0 and 1 were replaced by 0.08 (1/12) or 0.92 (11/12), respectively, because the SSRT cannot be calculated for SST_Uncancelled_Rates of 0 or 1. For the Transcriptional Task, we counted the number of transcribed words and the number of mistakes in addition to the depletion scores.

##### 4.1.4.2 Statistical analysis

R studio (Version 1.4.1106) was used to pre-process the data and SPSS (Version 26) was used for statistical analysis. A mean split was used for MSRS score to create a high and low MSRS group. In addition, initial perusal of the data showed large individual differences in the (perceived) depletion scores, also within the two intervention groups. Not all participants of the EDG were depleted, while participants in the CG were not always nondepleted. Because our interest is in the effects of the actual level of depletion (rather than whether or not participants had undergone the ego-depletion treatment), we also used mean split on the depletion scores to create a high and a low ego-depletion group (i.e., high and low ED group). Next, the SST_Uncancelled_RT and SST_Go_RT in both the baseline test and post-intervention test were compared for each participant. If the SST_Uncancelled_RT was longer than the SST_Go_RT in either the baseline test or post-intervention test, then the independence assumptions of the horse-race model were violated, and the participant would be excluded. No participant, however, was excluded for this reason. In addition, if a participant’s SST_Uncancelled_Rate was outside 15% and 85% range they would have been excluded as well from further analyses (Chen et al., 2009). Yet, this did not apply to any participant either. However, one participant in the CG was excluded because of very low SST_Go_Correct_Rate (i.e., 55.6%).

The number of transcribed words, the number of mistakes and the depletion scores of the high and low ED groups were compared using the independent *t*-tests. Next, the SST_Go_RT, SST_Go_Correct_Rate, SSRT, and SST_Uncancelled_Rate were submitted to a separate 2 (MSRS group: high MSRS, low MSRS) by 2 (ego-depletion group: high ED, low ED) by 2 (test: baseline, post-intervention) ANOVAs with repeated measures on the last factor. The simple main effect analysis was used to follow up significant effects. Because slowing down the go response might distort the estimation of SSRT (Band et al., 2003; Boehler et al., 2012; Verbruggen et al., 2013), the response slowing was measured by subtracting the CRTT_Go_RT from the SST_Go_RT. Specifically, if a significant three-way interaction on SSRT were to be revealed, response slowing would be submitted to a similar three-way ANOVA. Partial eta-squared ($\eta_{p}^{2}$) was calculated for ANOVA to estimate the effect size with values of 0.01, 0.06, and 0.14 considered as small, medium, and large, respectively. Cohen’s *d* was calculated for the independent *t*-test to estimate the effect size with values of 0.2, 0.5, and 0.8 considered as small, medium, and large, respectively.

### 4.2 Results

#### 4.2.1 Group assignment

According to the mean depletion scores (*M* = 7), 19 participants were assigned to the high ED group (*M* = 8.90, SD = 0.94) and 20 participants were assigned to the low ED group (*M* = 5.75, SD = 1.07). Table 4 shows that the high ED group had significant lower number of transcribed words compared to the low ED group, *t*(37) = 2.41, *p* = 0.02, *d* = 0.77, and the depletion scores were significantly higher for the high ED group compared to the low ED group, *t*(37) = −9.75, *p* < 0.001, *d* = −3.12. However, no significant difference between the two groups was found on the number of mistakes. According to the mean MSRS score (*M* = 42.77, SD = 8.53), 20 participants were assigned to the high MSRS group (*M* = 49.40, SD = 4.48) and 19 participants were assigned to the low MSRS group (*M* = 35.79, SD = 5.66).

**TABLE 4:** Descriptives and statistics of the transcriptional task in Experiment 3.

	High ED	Low ED	*t*(37)	*p*	*d*
The number of transcribed words	566.68 (16.40)	576.95 (9.42)	2.41	**0.02**	0.77
Depletion scores	8.90 (0.94)	5.75 (1.07)	−9.75	**<0.001**	−3.12
The number of mistakes	4.32 (8.29)	3.75 (11.91)	−0.17	0.87	−0.06

The number in parenthesis indicates SD. Number in bold refers to *p* < 0.05.

#### 4.2.2 SST

**FIGURE 5:**
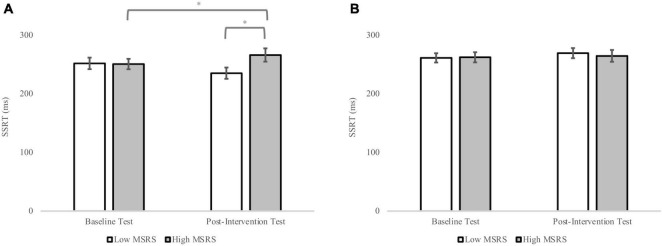
The SSRT in the SST for the low and high MSRS groups with high and low ego-depletion in the baseline and post-intervention test. **(A)** SSRT for the high and low MSRS group with high ego-depletion in the baseline and post-intervention test; **(B)** SSRT for the high and low MSRS group with low ego-depletion in the baseline and post-intervention test. Error bar represents the SEM. *p < 0.05.

There were no significant main effects nor any significant two-way interactions with SSRT (Table 5). However, a significant MSRS group by ego-depletion group by test interaction was found, *F*(1,35) = 4.71, *p* = 0.04, $\eta_{p}^{2}=0.12$. The simple main effect analyses indicated that the high MSRS group with high ego-depletion showed longer SSRT in the post-intervention test compared to the baseline test, *p* = 0.05 (Figure 5A). By contrast, the low MSRS group with high depletion scores did not show a significant difference between the two tests, *p* > 0.05. Additionally, neither the high MSRS group nor the low MSRS group with low depletion scores showed significant differences between baseline and post-intervention tests (Figure 5B), both *p*s > 0.05. The simple main effect analysis also revealed that in the post-intervention test the high MSRS group with high ego-depletion showed longer SSRT compared to the low MSRS group with high ego-depletion, *p* = 0.05 (Figure 5A). However, no difference in SSRT between the high and low MSRS group with high depletion scores in the baseline test was found, *p* > 0.05. Furthermore, no difference in SSRT between high and low MSRS group with low depletion scores in the baseline or post-intervention test was found, both *p*s > 0.05.

**TABLE 5:** Statistics of the stop trials in Experiment 3.

	SSRT			SST_Uncancelled_Rate		
	*F*(1,35)	*p*	* $\eta_{p}^{2}$ *	*F*(1,35)	*p*	* $\eta_{p}^{2}$ *
Test	0.28	0.60	0.01	0.66	0.42	0.02
Ego-depletion group	2.27	0.14	0.06	0.03	0.88	<0.01
MSRS group	0.54	0.47	0.02	0.10	0.75	<0.01
Test × ego-depletion group	0.45	0.51	0.01	0.81	0.37	0.02
Test × MSRS group	2.32	0.14	0.06	6.98	**0.01**	0.17
Ego-depletion group × MSRS group	0.89	0.35	0.03	4.16	**0.05**	0.11
Test × ego-depletion group × MSRS group	4.71	**0.04**	0.12	1.09	0.30	0.03

Number in bold refers to *p* < 0.05.

**FIGURE 6:**
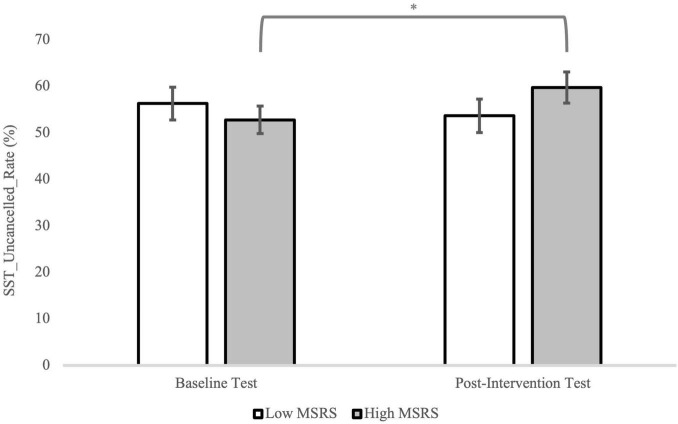
The SST_Uncancelled_Rate in the SST for the low and high MSRS groups in the baseline and post-intervention test. Error bar represents the SEM. *p < 0.05.

**FIGURE 7:**
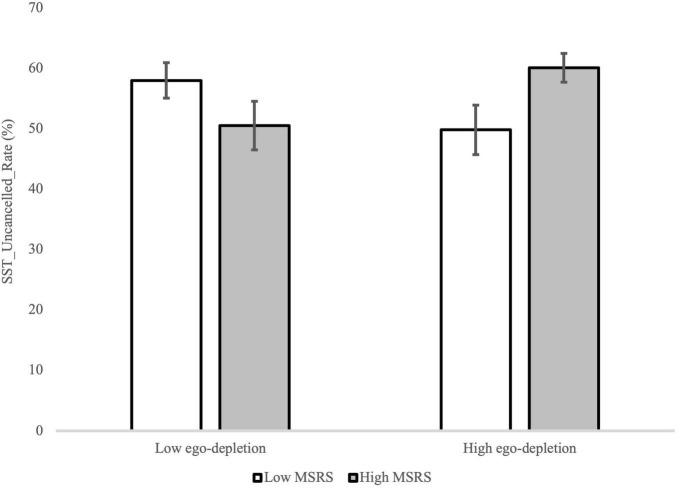
The SST_Uncancelled_Rate in the SST for the low and high MSRS groups and low and high ego-depletion group. Error bar represents the SEM.

The ANOVA for SST_Uncancelled_Rate showed a significant MSRS group by test interaction, *F*(1,35) = 6.98, *p* = 0.01, $\eta_{p}^{2}=0.17$. The simple main effect analyses indicated that for the high MSRS group the SST_Uncancelled_Rate was higher in the post-intervention test compared to the baseline test, *p* = 0.02, while for the low MSRS group no difference between the two tests was found, *p* = 0.21 (Figure 6). In addition, a significant MSRS group by ego-depletion group interaction was found, *F*(1,35) = 4.16, *p* = 0.05, $\eta_{p}^{2}=0.11$. However, the simple main effect analyses did not identify significant differences (Figure 7).

For SST_Go_RT, SST_Go_Correct_Rate and response slowing, no main effects, two-way interactions and three-way interactions were found after performing three-way ANOVAs (Table 6), Table 7 displays the descriptives results.

**TABLE 6:** Statistics of the go trials in Experiment 3.

	SST_Go_RT			SST_Go_Correct_Rate			Response slowing		
	*F*(1,35)	*p*	* $\eta_{p}^{2}$ *	*F*(1,35)	*p*	* $\eta_{p}^{2}$ *	*F*(1,35)	*p*	* $\eta_{p}^{2}$ *
Test	0.45	0.51	0.01	0.40	0.53	0.01	0.45	0.51	0.01
Ego-depletion group	1.77	0.19	0.05	2.22	0.15	0.06	0.68	0.42	0.02
MSRS group	3.62	0.07	0.09	0.35	0.56	0.01	2.91	0.10	0.08
Test × ego-depletion group	0.06	0.82	<0.01	2.39	0.13	0.06	0.06	0.82	<0.01
Test × MSRS group	0.29	0.59	0.01	0.24	0.63	0.01	0.29	0.59	0.01
Ego-depletion group × MSRS group	0.08	0.78	<0.01	0.93	0.34	0.03	1.81	0.19	0.05
Test × ego-depletion group × MSRS group	0.32	0.58	0.01	<0.01	0.97	<0.01	0.32	0.58	0.01

**TABLE 7:** Descriptives of the go trials in Experiment 3.

	High MSRS				Low MSRS			
	High ED		Low ED		High ED		Low ED	
	Baseline	Post-intervention	Baseline	Post-intervention	Baseline	Post-intervention	Baseline	Post-intervention
SST_Go_RT (ms)	403.68 (33.06)	401.91 (37.04)	388.43 (32.67)	379.95 (33.60)	422.63 (58.39)	420.69 (33.37)	409.03 (35.57)	409.86 (36.35)
SST_Go_Correct _Rate (%)	98.15 (4.20)	98.84 (1.63)	99.31 (0.96)	98.73 (1.39)	96.43 (7.47)	97.49 (4.80)	99.46 (0.62)	99.31 (1.13)
Response slowing (ms)	68.99 (66.08)	67.23 (71.02)	81.85 (35.31)	73.37 (42.53)	124.75 (63.90)	122.82 (35.42)	83.74 (55.72)	84.58 (56.38)

The number in parenthesis indicates SD.

### 4.3 Discussion

In Experiment 3, we tested whether reducing the capacity for conscious movement investment using an ego-depletion procedure impaired movement inhibition, and whether such effect would be enlarged for participants with a strong inclination for conscious movement investment. We anticipated that a decreased capacity for conscious movement investment due to ego-depletion would slow down inhibition (i.e., increase SSRT), and especially among participants who have a higher inclination for conscious movement investment. Indeed, we found that, for participants who felt highly depleted, the SSRT was longer for those with a high MSRS score than for those with a low MSRS score in the post-intervention test. This difference occurred because highly depleted participants with a high MSRS score prolonged their SSRT after the ego-depletion procedure, while highly depleted participants with a low MSRS score did not show a significant change in SSRT. This aligns with our hypothesis that a decreased capacity for conscious movement investment can impair movement inhibition, but only in participants who have a high inclination for conscious movement investment. This is consistent with previous observations that suggest a positive relationship between the degree of conscious movement investment and movement inhibition (Beilock and Gray, 2012; Park et al., 2020).

In addition, we found that individuals with a relatively high inclination for conscious movement investment showed a higher uncancelled rate in the post-intervention test in comparison to the baseline test (cf. Experiment 2). This increase in failing to inhibit movement, however, was present irrespective of the feeling of depletion. Since prior studies have shown that the speed of the go process might affect the estimation of SSRT (Band et al., 2003; Boehler et al., 2012; Verbruggen et al., 2013), it might be argued that the higher uncancelled rate and longer SSRT in the post-intervention test for participants with a high inclination for conscious movement investment (and who feel highly depleted) may be attributed to participants being more strongly biased towards fast responding over successful stopping compared to the baseline test. However, we did not find any indication for response slowing (Table 6), suggesting that the longer SSRT in the post-intervention test compared to the baseline test for individuals with a high MSRS score and high depletion scores was not because of the strategy that they used (i.e., a different trade off) in two tests.

Together, these findings indicate that a purported decrease in capacity for conscious movement investment after the ego-depletion procedure can deteriorate the ability for movement inhibition, particularly in participants that are predisposed to consciously invest in movement performance.

## 5 General discussion

There is a strong consensus that conscious involvement in well-developed motor skills normally impairs motor performance (Beilock et al., 2002; Chell et al., 2003; Jackson et al., 2006, 2013). By contrast, Beilock and Gray (2012) and Park et al. (2020) signalled that conscious attention to movement may enhance movement inhibition, instead of disrupting it. They reported that individuals who likely have high degrees of conscious movement investment (i.e., because they are less skilled or have a high predisposition for conscious movement investment) showed better ability to stop than individuals with low degrees of conscious movement investment. However, the evidence presented remained circumstantial, because it was deduced from (purported) individual or group differences in conscious movement investment rather than its direct manipulation. Hence, the present study further examined the relationship between conscious movement investment and movement inhibition, not only by exploring (purported) differences in individuals’ conscious movement investment (Experiment 2), but also by comparing movement inhibition under conditions with distinct constraints on conscious movement investment (Experiments 1 and 3). Two hypotheses were formulated based on prior studies: (1) when constraints are imposed that alter conscious movement investment (i.e., by directing attentional focus away from the movement, Experiment 1) or the capability to consciously invest the movement (i.e., by limiting the available resources for conscious movement investment via ego-depletion, Experiment 3) then inhibition would be degraded; and (2) these effects would be more pronounced for individuals with a high inclination for conscious movement investment.

The two hypotheses were substantiated, but only in Experiment 3. Participants with a high propensity for conscious movement investment needed more time to stop their response when they felt mentally depleted, while no slowing down of inhibition was found among participants that felt less depleted and/or had a low inclination for conscious movement investment. This indicates that conscious movement investment can affect inhibition but only under a particular set of interacting constraints, as per Experiment 3. These conditions were seemingly not met in Experiments 1 and 2. The findings align with previous observations (Beilock and Gray, 2012; Park et al., 2020) and strengthen but nuance previous interpretations. In none of the three experiments did a direct comparison between individuals with (purported) high and low degrees of conscious movement investment reveal differences in movement inhibition, except when the task was demanding relative to available resources. In other words, neither the GNG task, even if attention was explicitly directed to the movement, nor the more difficult SST of themselves required sufficient conscious movement investment to affect inhibition. Only, when also the capacity for conscious movement investment was reduced, by depleting resources for self-control, inhibition slowed down. In other words, the degree of conscious movement investment can help to stop fast, but the effect only emerges from the dynamic interaction of individual (e.g., skill level, inclination for conscious movement investment, current resources for conscious movement investment) and task constraints (e.g., difficulty of the task).

In this respect, it is to be expected that more complex movement tasks with a higher number of degrees of freedom than the current button-press movement may show a more pronounced relationship between (the inclination for) conscious movement investment and movement inhibition, as was the case in Beilock and Gray (2012) golf putting task. Hervault et al. (2021) proposed that the time to stop or inhibit scales with the number of separate degrees of freedom to be controlled. Thus, future studies should consider using more complex movement tasks involving higher number of degrees of freedom.^5^ In fact, in a follow-up study, the present authors explored the relationship between (the inclination for) conscious movement investment and the inhibition of a golf putting stroke (You et al., 2024). This showed a relationship between conscious movement investment and stopping the golf stroke, but also suggested that this relationship is perhaps indirect via a change in movement kinematics.

Next to increasing the theoretical understanding of the relationship between conscious movement investment and motor inhibition, uncovering the constraints that affect inhibition is also of practical importance, for instance in sports performance. Athletes in open skilled sports, such as table tennis and baseball, often need to stop or adjust their planned movements to a constantly changing environment. Especially with severe time constraints, fast inhibition may bolster sport performance. There is an increasing amount of studies addressing how different degrees of conscious investment during practice or in preparation of the match shapes motor control and performance in competition and under pressure (e.g., Masters, 1992; Masters and Maxwell, 2008). This has led to proposals to preferably rely on training methods that reduce conscious investment. Yet, it is unclear whether these findings generalize to stopping or adjusting of movements. Clearly, it is of high practical relevance to shed further light on these issues.

Although the current study provides additional insights compared to previous studies, it is important to acknowledge some limitations. First, although we also manipulated the demand for conscious movement investment, and thus did not fully rely on presumed interindividual differences in conscious movement investment, it remains imperative for providing more direct evidence that subsequent research finds ways to gauge participants’ actual levels of conscious movement investment, such as using verbal knowledge protocols to measure the amount of explicit movement-knowledge accumulated. Second, all three studies were conducted online because of the restrictions associated with the COVID-19 pandemic. While online research allowed researchers to continue their work during the pandemic, Finley and Penningroth (2015) argued that online research is less-controlled compared to laboratory-based research. Thus, replicating the findings in more controlled laboratory setting is important.

## 6 Conclusion

To conclude, the present research provides some evidence that increased conscious movement investment is associated with enhanced inhibition, and thus that conscious investment in movement is not generally debilitative. However, we also show that the effects of conscious investment in movement always emerge from the interaction with other constraints, such as, task difficulty, skill level, the inclination for conscious investment and the available resources.

## Data availability statement

The raw data supporting the conclusions of this article will be made available by the authors, without undue reservation.

## Ethics statement

The studies involving humans were approved by the Scientific and Ethical Review Board (VCWE) of the Faculty of Behavioural and Movement Sciences, Vrije Universiteit Amsterdam. The studies were conducted in accordance with the local legislation and institutional requirements. The participants provided their written informed consent to participate in this study.

## Author contributions

YY: Conceptualisation, Data curation, Formal analysis, Methodology, Software, Supervision, Validation, Visualisation, Writing – original draft, Writing – review & editing. W-CW: Conceptualisation, Data curation, Formal analysis, Investigation, Methodology, Visualisation, Writing – original draft, Writing – review & editing. GS: Conceptualisation, Data curation, Formal analysis, Investigation, Methodology, Visualisation, Writing – original draft, Writing – review & editing. JK: Conceptualisation, Project administration, Supervision, Writing – original draft, Writing – review & editing.

## Footnotes

1 The concept of conscious movement investment is adapted from the theory of reinvestment (Masters and Maxwell, 2008). The theory of reinvestment holds that automatized movements are disrupted if a skilled performer re-uses or re-invest task-relevant declarative or explicit movement-related knowledge to monitor and control a movement. This especially happens under stressful circumstances (Masters, 1992). With conscious movement investment, we refer to the same process of using explicit movement-related knowledge to monitor and control a movement but in a more general way, also including novice performers deliberately monitoring and controlling movements in non-stressful circumstances.

2 The reversal of causality relative to Beilock and Gray (2012) and our arguments.

3 In the pilot work, we found the SST to be a more fatiguing task and decided to run it first. However, while structuring the paper, we determined that presenting it in the current sequence was better.

4 To reduce the number of trials in the SST for both tests, we adopted a different SST procedure (Chen et al., 2009; Wang et al., 2013). This resulted in the visual stimuli and procedures for the CRTT and SST in Experiment 3 being different from those in Experiment 2.

5 The present button-press tasks were chosen because they allowed us to continue experimental investigations during COVID-19 lock downs. Consequently, participants participated from home. The resulting impoverished interactions between experimenter and participants may have unknown effects on participants’ task performance.

## Appendix

**Appendix:** The abbreviations of dependent variables.

Abbreviations	Meanings
CG	Control group
CRTT	Choice reaction time task
CRTT_Correct_Rate	The ratio of correct responses to all responses in the CRTT
CRTT_Go_RT	The mean reaction time on all go trials in the CRTT
EDG	Ego-depletion group
GNG task	Go/no-go task
Go_Correct_RT	The mean reaction time for correct go responses in the GNG task
Go RTV	The variability in reaction time on go trials in the GNG task
High ED group	High ego-depletion group
Low ED group	Low ego-depletion group
MSRS	Movement-Specific Reinvestment Scale
No-Go_Commission_Rate	The ratio of commission errors to all responses on no-go trials in the GNG task
SSD	Stop-signal delay
SSRT	Stop-signal reaction time
SST	Stop-signal task
SST_Go_Correct_Rate	The ratio of correct responses to all responses on go trials in the SST
SST_Go_RT	The mean reaction time on all go trials in the SST
SST_SSD	Mean stop-signal delay
SST_Uncancelled_RT	The mean reaction time on stop trials where participants failed to stop or cancel the response in the SST
SST_Uncancelled_Rate	The ratio of uncancelled responses to all responses on stop trials in the SST
Go_Correct_Rate	The ratio of correct responses to all responses on go trials in the GNG task
